# Perceived parental rejection mediates the effects of previous maltreatment on emotional and behavioural outcomes in Chinese adolescents whereas mental illness has no moderating effect

**DOI:** 10.4102/sajpsychiatry.v23i0.1073

**Published:** 2017-06-30

**Authors:** Bo Xiao, Jianbo Liu, Jingbo Gong, Xuerong Luo

**Affiliations:** 1 Mental Health Institute, The Second Xiangya Hospital, Central South University, China; 2 The China National Clinical Research Center for Mental Health Disorders, National Technology Institute of Psychiatry, Key Laboratory of Psychiatry and Mental Health of Hunan Province, China; 3 Department of Applied Psychology, Traditional Chinese Medicine University of Hunan, China

## Abstract

**Objective:**

This study assessed the mediating role of perceived parental rejection in the relationship between childhood maltreatment experience and behavioural problems in Chinese adolescents.

**Methods:**

A total of 2484 adolescents (1305 males and 1179 females; aged 12–16 years) from Hunan Province, China, participated in the study. Behavioural problems, parental rejection scores and child abuse experiences were evaluated by the Child Behavior Checklist (parental version), the Memories of Parental Rearing Behavior Scale and the Childhood Trauma Questionnaire, separately. Mediating effects were examined by structural equation modelling using Amos 20 software.

**Results:**

The study found that perceived maternal rejection partially mediated the association between abuse and internalising behaviours in the male cohort, whereas perceived father’s rejection partially mediated this association in the female cohort. However, mental illness had no moderating effect on these relationships.

**Conclusion:**

These results are consistent with the literature on maltreatment and parent-child relationships and provide empirical support for the view that emotional and behavioural problems related to perceived parental rejection underlie the development of psychosocial problems in adolescents.

## Introduction

Childhood maltreatment can be defined as any act or series of acts of commission or omission by a parent or other caregiver, in the context of a relationship of responsibility, trust or power, which actually or potentially harms a child’s health, survival, development or dignity.^1^ As a type of adverse childhood experience, maltreatment (physical abuse, sexual abuse, emotional abuse and/or neglect) can lead to the development of psychiatric problems, such as depression and anxiety, panic disorder, social phobia, sleep disorders, suicide attempts, substance abuse, post-traumatic stress disorder and personality disorder, during adolescence or later in adult life.^2,3,4^ Victims of maltreatment during childhood are also at risk of increased severity of psychological disorders later in life, compared with individuals who were not abused by their parents or other caregivers.

Child maltreatment has become a global health problem, despite increased awareness of the high prevalence of such abuse. Child maltreatment rates remain at unacceptably high levels worldwide and pose serious risks of behavioural problems and mental illness in children and adolescents. The majority of studies of childhood maltreatment have been conducted in developed countries.^5,6^ In China, childhood abuse has not been given sufficient attention in the past because of the influence of traditional cultural norms,^7,8^ and nationwide epidemiological survey data are lacking. However, increasing evidence shows that child abuse remains a problem in China, and the mental health status of Chinese children and adolescents cannot be ignored.^9,10^ Our research team recently documented a high overall point prevalence of mental disorders in Chinese children and adolescents (9.74% in children aged 6–16 years; 2015, unpublished), which confirms the need to explore potential associations of such conditions with child maltreatment.

### Outcomes of childhood maltreatment

Several previous studies have demonstrated that children who have experienced maltreatment present more internalising and externalising problems than do those who have not been maltreated.^11^ These emotional and behavioural problems include anxiety, psychosomatic complaints, social regression, withdrawal, irritability and depression, antisocial conduct, delinquency, aggression and hyperactivity. Adolescence is a period of, especially, increased vulnerability to the development of behavioural problems,^12,13^ which are diverse and can be classified broadly as externalising or internalising behaviour.

Not all children who have been exposed to abuse present externalising or internalising behaviour, and not all such children show signs of damage to their mental health as they age. Childhood abuse can lead to the manifestation in later life of diverse clinical symptoms and a wide range of disease severity. Although the existence of direct relationships between childhood abuse experiences and later outcomes is recognised universally, potential mediators of this complex relationship are not well understood. Many variables can likely help to explain such relationships.

### Effects of negative parental practices: Parental rejection

One important explanatory variable may be parental rearing practices, which encompass parenting attitudes, behaviours and values regarding the development of children.^14^ As the family is the main setting for children’s socialisation, parents’ rearing behaviours are essential for the development of children’s behaviour. Several studies have investigated the influence of parenting style on the behaviour of children and adolescents.^15^ Negative parenting practices, such as parental rejection, are associated with the development of internalising symptoms in children.^16,17^ The mechanisms underlying this association are not understood fully; one possible explanation is that perceived parental rejection delays the development of attachment and renders a child unwilling to accept parental values and beliefs.^18^ Some psychiatric research has associated parental rejection with aggression, depression and anxiety symptoms in children.^19,20^

Children benefit when parents have safe, stable and nurturing relationships. In contrast, children exposed to poor family relationships and negative parenting behaviours are more likely to develop more internalising and externalising problems.^21^

In China, traditional cultural norms lead to the tendency of parents to use severe punishment, rejection, overprotection and other negative parenting practices, including extreme behaviour in some cases, such as physical abuse. In addition, because of socioeconomic factors, the number of ‘left-behind’ children (those aged < 18 years who have been left behind at their original residences for ≥ 6 months while one or both parents migrate to other areas to work) has increased annually. The lack of positive parenting had produced severe mental health issues and social problems in affected youth in China.^22^

### Maltreatment, rejection and emotional/behavioural problems

The relationship between parenting practices and children’s behavioural and emotional problems is bidirectional. Maltreatment and its outcomes affect the development of parenting attitudes and practices. For example, negative behaviours of a child exposed to physical abuse (e.g. screaming, resistance, hyperactivity, aggression, withdrawal) will lead to more perceived parental rejection and punishment. Some scholars have argued that chronic parental rejection should be considered a core aspect of childhood emotional maltreatment.^23,24^

Fortunately, some interventional measures have been shown to have positive effects on behavioural problems and mental disorders in children and adolescents. Behaviourally oriented parenting programs and parent-child interaction therapy are known to effectively increase parenting skills, change parenting styles, and decrease the occurrence and severity of behavioural problems in children.^25^

In summary, the associations of previous maltreatment and children’s perceptions of parental rejection with their presenting symptoms and behaviour remain incompletely understood. The mechanisms by which these complex factors interact in the development of mental disorders in children and adolescents are unknown. A recent study in Japan showed that perceived parental rejection mediated the relationship between the 5-HTTLPR genotype and impulsive behaviour in Japanese adults,^26^ and another study in Canada revealed that trauma symptoms fully mediated the associations between maltreatment and internalising and externalising behaviours.^27^ However, little research has examined the relationship between childhood maltreatment, particularly perceived parental rejection, and later manifestation of internalising and externalising problems. Hay^28^ found that parental rejection was related to internalising behaviours in girls more than in boys. In the United States, Moylan et al.^29^ found higher levels of internalising behaviours among maltreated female adolescents, whereas maltreated male adolescents displayed more externalising problems. These findings imply the existence of distinct gendered pathways linking child abuse to later outcomes; the exploration of such differences is important for prevention and intervention strategies.^30^

### Study objectives and hypotheses

The overall objective of this research was to improve our understanding of emotional and behavioural problems in children who have been exposed to maltreatment and parental rejection in the home environment. The study explored three main research questions:

1. Does perceived parental rejection mediate the relationship between previous maltreatment and children’s later internalising and externalising behaviours?
2. If present, does this process differ according to children’s gender?
3. Are the associations of child maltreatment and perceived parental rejection with later outcomes in adolescents moderated by mental disorder status?

We hypothesised that the relationship between child maltreatment and later behaviours would be mediated by rejection from the mother or the father. We also hypothesised that this relationship would differ between male and female children and would be moderated by the presence of mental disorders in children.

## Subjects and methods

### Participants and procedures

This study was conducted between 31 June 2013 and 31 May 2015 as part of a larger study of the mental health of children in China. The minimum sample size was calculated according to the national prevalence of children with schizophrenia in China, using data from the sixth national census (2010)^31^ on the overall population ratio of children aged 6–16 years. A total of 18 778 children and adolescents from the Changsha and Yiyang regions of Hunan Province were enrolled in the larger epidemiological study. Eight urban schools and eight rural schools in each region were selected randomly using Excel software. All students from 32 primary and middle schools were included in the study.

**FIGURE 1:**
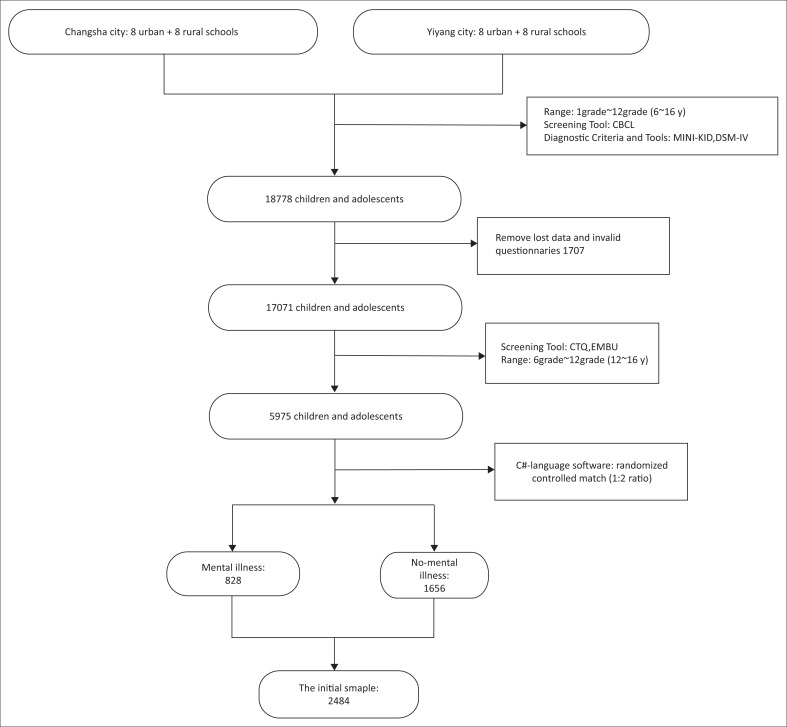
The flowchart of detailed investigative steps.

Questionnaire packets were distributed to children and adolescents (aged 6–16 years) in first through 12th grades and to their parents or other caregivers. Parents or caregivers completed the Child Behavior Checklist (CBCL) for screening, followed by one-to-one administration of the Mini-International Neuropsychiatric Interview for Children and Adolescents (MINI-KID) to students. When results were positive, psychiatrists made clinical diagnoses using the criteria of the Diagnostic and Statistical Manual of Mental Disorders, 4th edition (DSM-IV).^32^ Some of these children and adolescents (aged 12–16 years) completed the Childhood Trauma Questionnaire (CTQ) and the Memories of Parental Rearing Behavior or Egma Minnen av Barndom Uppforstran (EMBU) Scale. All children and adolescents were interviewed at school. Figure 1 is a flowchart of the study procedure.

### Diagnostic criteria and tools

The MINI-KID^33^ is a short, structured clinical diagnostic interview designed to assess the presence of psychiatric disorders codified in the DSM-IV and the International Classification of Diseases, 10th Revision, in children and adolescents. The Chinese children’s version of the MINI-KID has demonstrated high diagnostic sensitivity and has been shown to be suitable for comprehensive psychiatric diagnosis in children and adolescents.^34^

### Indicator variable measures

#### Childhood Trauma Questionnaire

The CTQ^35^ is a 28-item, retrospective, self-report questionnaire designed to assess 5 types of negative childhood experience (emotional, physical and sexual abuse; emotional and physical neglect). Items are rated on a 5-point Likert-type scale ranging from 1 (never true) to 5 (very often true). The CTQ has demonstrated adequate sensitivity and specificity in relation to therapist’s ratings of maltreatment.^36^ The Chinese version of the CTQ, used in this study, has shown good reliability and validity in Chinese adolescents and adults. In this study, the Cronbach’s alpha value of the CTQ was 0.89.

#### Child Behavior Checklist

One-hundred-thirteen items from the behavioural problems’ section of the standardised Chinese parental version of the CBCL^37^ were used in this study. The CBCL covers important aspects of child and adolescent mental health. Parents or other caregivers are instructed to use a 0–1–2 scale to score items. The sum of individual item scores is the total behaviour-problem score; the calculation of different subscale scores is also possible. The 113 items used in this study measure a variety of psychosocial behaviours, which were grouped into two global scales: internalising (anxiety or depression, withdrawal, schizophrenia and somatic complains) and externalising (delinquency and aggression) behaviours. In the current study, this questionnaire was completed by children. The Cronbach’s alpha values were 0.82 for internalising problems and 0.87 for externalising problems. Raw scores for these two scales were used in the present analyses.

#### Memories of Parental Rearing Behavior Scale

The EMBU Scale for Children^38^ was used in this study to assess children’s perceived parental rejection. This instrument consists of 81 items scored on a 4-point scale ranging from 1 (no or never) to 4 (yes or always) for the father and the mother separately. Seventeen items were used in this study to measure rejection. Cronbach’s alpha values for the EMBU Scale ranged from 0.69 to 0.91. The Chinese version of this scale, used in this study, has shown good psychometric properties.^39^

### Data analysis

The consistency of diagnostic results obtained by the researchers was assessed, yielding kappa values of 0.85–0.91. Descriptive statistics were calculated for study variables. Spearman and Pearson correlation analyses (for dichotomous and continuous variables, respectively) and multiple linear regression analyses were performed to assess relationships among the variables of interest. Structural equation models were constructed using SPSS Amos 20^40^ to evaluate the mediating effects of the variables specified by models with path diagrams. The relationships among the predictor (child maltreatment), mediators (rejection by mother and/or father) and children’s internalising or externalising behavioural problems were assessed. Amos 20 calculates all measures that capture model evaluation, which were selected based on the following: the minimum value of sample discrepancy divided by its degree of freedom (smaller values are preferable),^41^ the goodness of fit index (values > 0.95 are preferable), and the root mean square error of approximation (RMSEA) based on population discrepancy (values ≤ 0.05 indicate acceptable fit). Bootstrapping is an accurate way to assess indirect meditating effects,^42^ which were considered to be significant when the bias-corrected and accelerated bootstrapping confidence intervals (CIs) did not include 0 (95% CIs from 2000 bootstrap samples).To test the hypothesis that mental disease moderated the effects of parental rejection on the relationships between maltreatment and later outcomes, we performed heterogeneity tests. According to Altman and Bland,^43^ *z*-values exceeding ± 1.96 are significant (*p* < 0.05).

## Ethical considerations

All children and their parents or caregivers provided informed consent before study participation. The study complied with the provisions of the Declaration of Helsinki, and the protocol was approved by the Ethics Committee of the Second Xiangya Hospital, Central South University.

## Results

### Demographic data

Valid data were available for 17 071 participants, with a response rate of 90.9%. Among them, 5975 children fell within the age range for CTQ screening. They then underwent questionnaire-based screening and clinical diagnosis by psychiatrists. A consistency test was conducted to assess the diagnostic results obtained by the researchers, providing kappa values of 0.850.91. In total, 828 children and adolescents were diagnosed with mental illness (e.g. attention deficit hyperactivity disorder, anxiety, depression, schizophrenia). These adolescents were matched randomly with 1656 adolescents with no mental illness (1:2 ratio) using C# software. Thus, a total of 2484 individuals (1305 males, 1179 females; aged 12–16 years) participated in the present study. Table 1 presents descriptive statistics for the study population.

**TABLE 1:** Demographic characteristics of the two groups sample (the mental disorders group and the control group).

Variables	Mean (s.d.) or *N* (%)		*χ*^2^/*t*	*p*

	Mental disorders group *N* = 828	Control group *N* = 1656		
**Gender (Child characteristics)**				
Female	393 (47.5%)	786 (47.50)	0.000	1.000
Male	435 (52.50)	870 (52.50)	0.000	1.000
Age	13.57 (1.19)	13.57 (1.19)	0.000	1.000
**Nation**				
Han nationality	820 (99.03)	1645 (99.34)	0.663	0.416
Minority nationality	8 (0.97)	11 (0.66)	-	-
**Residence**				
City	259 (31.28)	328 (19.81)	46.863	< 0.001
Suburb	146 (17.63)	397 (23.97)	-	-
Rural	336 (40.58)	778 (46.98)	-	-
Other	87 (10.51)	153 (9.24)	-	-
Left behind children	182 (21.98)	214 (12.92)	2.805	0.094
**CTQ**−				
Emotional abuse	7.736 (3.401)	6.334 (2.037)	−10.918	< 0.001
Physical abuse	6.502 (2.943)	5.652 (1.721)	−7.686	< 0.001
Sexual abuse	5.639 (2.209)	5.382 (1.557)	−2.999	0.003
Emotional neglect	12.394 (5.434)	10.528 (4.723)	−8.419	< 0.001
Physical neglect	9.403 (3.576)	9.480 (3.265)	0.514	0.607
**Peer bullying victims**				
Frequently	828 (100.00)	826 (49.88)	623.253	< 0.001
Occasionally	-	230 (13.89)	-	-
Never	-	600 (36.23)	-	-
**Family characteristics (Family status)**				
Only-child family	263 (31.76)	381 (23.01)	22.037	< 0.001
No only-child family	565 (68.24)	1275 (76.99)	-	-
**Father’s age**				
20_~_29	2 (0.24)	6 (0.36)	2.764	0.429
30_~_39	224 (27.05)	416 (25.12)	-	-
40_~_49	574 (69.32)	1191 (71.92)	-	-
≥ 50	28 (3.38)	43 (2.60)	-	-
**Father’s cultural degree**				
Literacy	172 (20.77)	379(22.89)	25.136	< 0.001
Primary school	375 (45.29)	771 (46.56)	-	-
Middle school	191 (23.07)	263 (15.88)	-	-
High school	40 (4.83)	132 (7.97)	-	-
University plus	50 (6.04)	111 (6.70)	-	-
**Mother’s age**				
20_~_29	6 (0.72)	10 (0.60)	0.194	0.979
30_~_39	415 (50.12)	838 (50.60)	-	-
40_~_49	400 (48.31)	793 (47.89)	-	-
≥ 50	7 (0.85)	15 (0.91)	-	-
**Mother’s cultural degree**				
Literacy	195 (23.55)	410 (24.76)	6.940	0.139
Primary school	407 (49.15)	817 (49.34)	-	-
Middle school	169 (20.41)	295 (17.81)	-	-
High school	33 (3.99)	57 (3.44)	-	-
University plus	24 (2.90)	77 (4.65)	-	-
**Financial status**				
High-income	100 (12.08)	246 (14.86)	8.053	0.018
Middle-income	667 (80.56)	1326 (80.07)	-	-
Low-income	61 (7.37)	84 (5.07)	-	-

s.d., standard deviation.

### Correlations between variables of interest

Tables 2 and 3 present correlation coefficients for variables of interest in male and female adolescents, respectively. Parental (father or mother) rejection (as measured by the EMBU Scale) was correlated positively with emotional, physical or sexual abuse and neglect (as measured by the CTQ) and internalising and externalising behavioural problems (as measured by the CBCL) in the male and female samples (all *p* < 0.01). These correlations suggest that higher levels of parental rejection and childhood abuse resulted in higher levels of externalising and internalising behavioural problems.

**TABLE 2:** Correlation coefficients for all variables of interest in the male sample (*n* = 1305).

Variable	1	2	3	4	5	6	7	8	9	10	11	2	13	14	15
Age	1	0.023	−0.054*	−0.067*	0.036	0	0.048	0.068*	0.017	−0.058*	0.005	−0.032	0.109**	−0.048	0.02
Financial status†	0.022	1	−0.022	−0.027	0.086**	0.066*	0.097**	0.120**	0.089**	0.070*	0.037	0.052	0.04	0.068*	0.05
Father’s cultural degree†	−0.055*	−0.022	1	0.388**	0.057*	−0.025	0.02	−0.008	0.025	0.016	−0.018	−0.076**	−0.062*	−0.025	−0.007
Mother’s cultural degree†	−0.069*	−0.027	0.388**	1	−0.004	−0.053	−0.01	−0.013	−0.011	−0.043	−0.025	−0.057*	−0.058*	−0.036	−0.014
Left behind†	0.038	0.086**	0.057*	−0.004	1	0.129**	0.045	0.062*	0.083**	0.052	0.070*	0.078**	0.047	0.079**	0.077**
Mental disorders†	0	0.066*	−0.025	−0.053	0.129**	1	0.247**	0.241**	0.226**	0.202**	0.131**	0.150**	−0.005	0.643**	0.565**
Father rejection	0.048	0.114**	0.019	0.002	0.056*	0.235**	1	0.765**	0.338**	0.290**	0.128**	0.158**	0.080**	0.248**	0.217**
Mother rejection	0.068*	0.145**	−0.007	−0.012	0.076**	0.233**	0.765**	1	0.414**	0.342**	0.130**	0.165**	0.075**	0.242**	0.232**
Emotional abuse	0.017	0.099**	0.036	−0.015	0.107**	0.237**	0.338**	0.414**	1	0.657**	0.476**	0.282**	0.254**	0.226**	0.262**
Physical abuse	−0.058*	0.086**	0.007	−0.038	0.060*	0.190**	0.290**	0.342**	0.657**	1	0.487**	0.241**	0.223**	0.144**	0.167**
Sexual abuse	0.005	0.101**	0.046	0.033	0.101**	0.090**	0.128**	0.130**	0.476**	0.487**	1	0.172**	0.249**	0.106**	0.148**
Emotional neglect	−0.032	0.044	−0.062*	−0.044	0.069*	0.157**	0.158**	0.165**	0.282**	0.241**	0.172**	1	0.422**	0.138**	0.110**
Physical neglect	0.109**	0.034	−0.059*	−0.057*	0.058*	0.005	0.080**	0.075**	0.254**	0.223**	0.249**	0.422**	1	0.005	0.029
Externalising behaviour	−0.048	0.078**	−0.037	−0.047	0.087**	0.699**	0.248**	0.242**	0.226**	0.144**	0.106**	0.138**	0.005	1	0.829**
Internalising behaviour	0.02	0.062*	−0.006	−0.034	0.121**	0.614**	0.217**	0.232**	0.262**	0.167**	0.148**	0.110**	0.029	0.829**	1

† , Spearman correlation.

* *p* < 0.05;

** *p* < 0.01

**TABLE 3:** Correlation coefficients for all variables of interest in female sample (*n* = 1179).

Variable	1	2	3	4	5	6	7	8	9	10	11	12	13	14	15
Age	1	0.037	−0.046	−0.062*	0	0.004	−0.021	−0.031	−0.019	−0.059*	−0.027	−0.05	0.088**	−0.052	0.055
Financial status†	0.036	1	−0.043	−0.044	0.023	0.04	0.018	0.053	0.081**	0.034	0.068*	0.047	0.067*	0.027	0.060*
Father’s cultural degree†	−0.042	−0.043	1	0.440**	0.003	−0.02	0.044	0.066*	0.022	0.05	0.006	0.004	−0.073*	0.011	−0.004
Mother’s cultural degree†	−0.059*	−0.044	0.440**	1	−0.027	0.002	0.051	0.070*	0.026	0.072*	0.028	0.036	−0.049	−0.009	0.008
Left behind†	0	0.023	0.003	−0.027	1	0.103**	0.322**	0.270**	0.232**	0.200**	0.037	0.184**	−0.039	0.734**	0.734**
Mental disorders†	0.003	0.04	−0.02	0.002	0.103**	1	0.044	0.041	0.122**	0.066*	0.035	0.044	0.062*	0.090**	0.108**
Father rejection	−0.021	0.009	0.074*	0.063*	0.306**	0.041	1	0.735**	0.424**	0.298**	0.099**	0.228**	0.073*	0.292**	0.297**
Mother rejection	−0.031	0.03	0.067*	0.068*	0.270**	0.043	0.735**	1	0.517**	0.323**	0.094**	0.273**	0.061*	0.265**	0.246**
Emotional abuse	−0.019	0.117**	0.035	0.015	0.263**	0.126**	0.424**	0.517**	1	0.539**	0.340**	0.350**	0.199**	0.296**	0.265**
Physical abuse	−0.059*	0.076**	0.065*	0.055	0.174**	0.105**	0.298**	0.323**	0.539**	1	0.444**	0.278**	0.107**	0.180**	0.157**
Sexual abuse	−0.027	0.084**	0.017	0.013	0.033	0.075*	0.099**	0.094**	0.340**	0.444**	1	0.164**	0.189**	0.041	0.070*
Emotional neglect	−0.05	0.041	0.022	0.051	0.199**	0.055	0.228**	0.273**	0.350**	0.278**	0.164**	1	0.359**	0.187**	0.156**
Physical neglect	0.088**	0.063*	−0.074*	−0.054	−0.029	0.065*	.073*	0.061*	0.199**	0.107**	0.189**	0.359**	1	0.003	−0.016
Externalising behaviour	−0.052	0.026	0.016	−0.024	0.777**	0.087**	0.292**	0.265**	0.296**	0.180**	0.041	0.187**	0.003	1	0.857**
Internalising behaviour	0.055	0.051	0.006	−0.008	0.783**	0.114**	0.297**	0.246**	0.265**	0.157**	0.070*	0.156**	−0.016	0.857**	1

† , Spearman correlation.

* *p* < 0.05;

** *p* < 0.01

### Variables associated with externalising and internalising behaviours

Multiple linear regression analyses conducted for the male and female samples indicated that externalising behaviour (as measured by the CBCL) was affected significantly by mental disorders (males: β = 0.678, *p* < 0.001; females: β = 0.752, *p* < 0.001) and fathers’ rejection (males: β = 0.088, *p* < 0.001; females: β = 0.098, *p* < 0.001). These models explained 49.5% and 61.3%, respectively, of the variance in male and female adolescents’ externalising behavioural problems. Internalising behaviour (as measured by the CBCL) was affected significantly by five variables (mental disorder, mothers’ rejection, emotional abuse, sexual abuse and physical abuse) in the male sample and three variables (mental disorder, emotional abuse and fathers’ rejection) in the female sample (Table 4). Because no significant correlation was found among childhood abuse, parents’ rejection and externalising behaviour, no further structural equation testing was performed.

**TABLE 4:** Regression model for externalising and internalising behaviour (CBCL score).

Variable	Partial regression coefficient				Beta-value	*R*^2^	*R*^2^adj

	β/Coef	SE	*t*	*P*			
**Male: EB**							
Model	5.764	0.8	29.025	< 0.001	-	0.495	0.495
MD	18.335	0.548	33.465	< 0.001	0.678	-	-
FR(EMBU)	0.379	0.087	4.365	< 0.001	0.088	-	-
**IB**							
Model	1.732	0.893	1.939	0.053		0.398	0.396
MD	12.901	0.499	25.833	< 0.001	0.579	-	-
EA(CTQ)	0.43	0.118	3.631	< 0.001	0.112	-	-
MR(EMBU)	0.175	0.063	2.757	0.006	0.067	-	-
SA(CTQ)	0.343	0.127	2.711	0.007	0.069	-	-
PA(CTQ)	−0.299	0.121	−2.461	0.014	−0.073	-	-
**Female: EB**							
Model	5.764	0.800	7.209	< 0.001	-	0.613	0.613
MD	23.167	0.579	39.986	< 0.001	0.752	-	-
EA(CTQ)	0.557	0.106	5.227	< 0.001	0.098	-	-
**IB**							
Model	4.965	0.977	5.081	< 0.001	-	0.619	0.618
MD	24.011	0.607	39.554	< 0.001	0.757	-	-
EA(CTQ)	0.271	0.117	2.307	0.021	0.046	-	-
FR(EMBU)	0.249	0.113	2.206	0.028	0.045	-	-

CBCL, Child Behavior Checklist scale; MD, mental disorders; FR, father rejection; MR, mother rejection; EB, externalising behaviour; IB, internalising behaviour; EA, emotional abuse; PA, physical abuse; SA, sexual abuse; CTQ, Childhood Trauma Questionnaire; EMBU, Memories of Parental Rearing Behaviour scale or Egma Minnen av Barndom Uppforstran.

### Indirect mediation by parental rejection of the association between maltreatment and internalising problems

**FIGURE 2:**
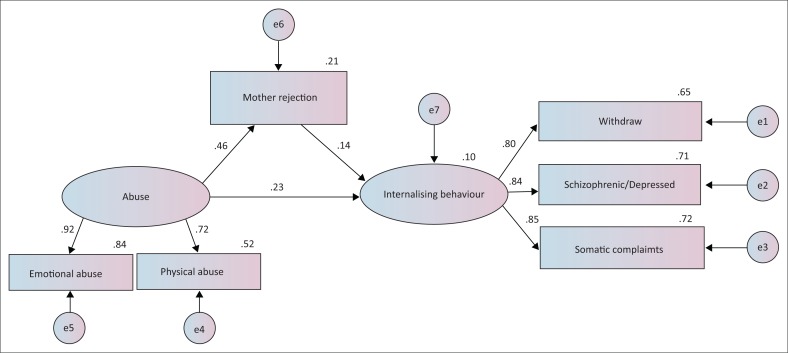
The partial mediation model in male sample (*n* = 1305).

**FIGURE 3:**
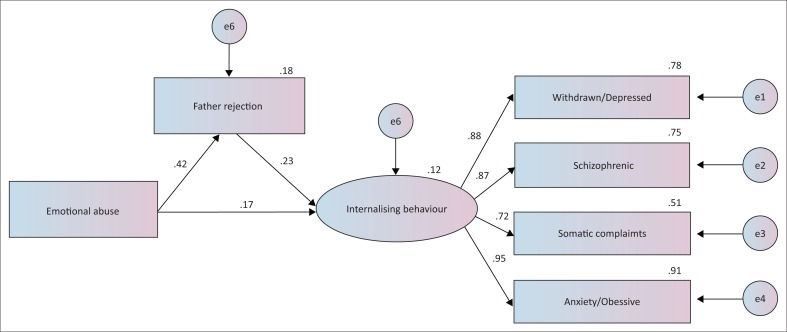
The partial mediation model in female sample (*n* = 1179).

The first model constructed to address the first research question by exploring the direct and indirect pathways by which parents’ rejection influenced children’s later behaviour did not fit the data from male adolescents well (χ^2^ = 128.070, df = 18, RMSEA = 0.068, comparative fit index [CFI] = 0.976, Tucker–Lewis index [TLI] = 0.958). According to these results, the immature and sexual abuse variables were removed because of low loading. Figure 2 presents the model with standardised estimates of path coefficients and factor loading for the latent construct. This model fit the data well (χ^2^/df = 2.794, RMSEA = 0.037, CFI = 0.996, TLI = 0.991; Table 5). The adjoining paths from child abuse (emotional and physical) through fathers’ rejection to internalising behavioural problems (withdrawal, depression, somatic complaints) were significant, and mothers’ rejection partially mediated the effects of exposure to abuse on children’s later internalising problems. The coefficients for the direct pathway from childhood abuse to internalising behavioural problems were also significant in the partial mediation model. Bootstrapping analysis revealed that mothers’ rejection partially mediated the relationship between abuse (emotional and physical) and internalising behaviour in the male sample (indirect model: 95% bootstrap CI [*n* = 2000] = 0.057–0.189). The final partial model explained 29.4% of the effect of previous maltreatment on internalising behaviours, 6.4% of which was indirect (through perceived mothers’ rejection). Figure 3 shows the model that fit the data from female adolescents well (χ^2^/df = 1.547, RMSEA = 0.022, CFI = 0.999, TLI = 0.998; Table 5). Bootstrapping analysis revealed that fathers’ rejection partially mediated the relationship between emotional abuse and internalising behaviour in the female sample (indirect model: 95% bootstrap CI [*n* = 2000] = 0.117–0.236). The final partial model explained 26.7% of the effect of previous maltreatment on internalising behaviour, 9.7% of which was indirect (through perceived fathers’ rejection).

**TABLE 5:** Comparisons among pathways from maltreatment to perceived rejection and internalising behaviour, using various measures of model fit.

Model	*X*^2^/df	GFI	RMSEA	CFI	TLI
**IB**					
Male	19.561/7	0.995	0.037	0.996	0.991
Female	12.374/8	0.996	0.022	0.999	0.998

IB, internalising behaviour; GFI, goodness of fit index; RMSEA, root mean square error of approximation; CFI, comparative fit index; TLI, Tucker–Lewis index.

### Moderating effects of children’s mental disorders

Mental disorders did not moderate the effects of parental rejection on the relationship between maltreatment and later outcomes (males: *z* = −1.296; females: *z* = −1.376). Thus, the model results shown in Figures 2 and 3 applied equally well to male and female children, respectively, irrespective of whether they have mental disorders.

## Discussion

In this cross-sectional study of 2484 Chinese adolescents, perceived paternal or maternal rejection was found to mediate the relationship between past maltreatment experience and internalising behavioural problems. The effects of maternal rejection appeared to be, especially, robust among males, whereas those of paternal rejection appeared to be, especially, powerful among females. We found no moderating effect of children’s mental disorders on these relationships.

Our results suggest that childhood maltreatment is a strong predictor of internalising behavioural problems in adolescence. Child abuse was not related significantly to externalising behavioural problems in male adolescents in this sample, in contrast to most previously reported findings. For instance, males have been reported to respond to maltreatment by exhibiting externalising behaviours, including aggressive behaviour, conduct problems and violence, whereas females have been found to be more likely to show internalising symptoms, such as depression, suicidal ideation or behaviours and eating disorders.^44^ The non-significant findings in the present study may be because of under-reporting by male adolescents. Increased paternal rejection (as measured by the EMBU Scale) was associated with more behavioural problems in boys, perhaps because fathers’ strict parenting styles undermine boys’ adaptive, social developmental and self-adjustment abilities.^45^ Fathers’ and mothers’ parenting styles had different effects on children’s behavioural problems in the present study, consistent with the results of previous research.^46,47^ These results may reflect the traditional Chinese childrearing style of ‘strict father, loving mother’, which leads to differences in children’s perceptions of parental rearing patterns.^22^ In addition, maternal rejection seemed to play an important role in male adolescents’ behaviour, whereas paternal rejection appeared to have a greater effect on females. More studies are needed to examine the relationships between gender and perceived parental rejection.

Children’s mental illness showed no effect on the relationships examined in this study. Results of previous studies of emotional and behavioural problems in children have indicated that such effects on internalising problems may not be detectable in cross-sectional samples. Considering the development of psychopathology perspective would propose, children have different experiences of the same event, depending on the level of functioning.^48^ In addition, the nature and timing of experiences influence children’s construction of their meaning, resulting in differences in response and adaptation to situations. In other words, the ways in which children respond to maltreatment may lead to real developmental differences, which need to be assessed longitudinally.

In summary, the findings of this study provide support for the hypothesis that childhood maltreatment predicts internalising behavioural problems via parental rejection, thereby shedding light on the mechanisms that contribute to the development of behavioural problems in Chinese adolescents. Overall, it would be beneficial to have a clearer picture of the role that parental rejection plays in the developmental trajectories of individuals who have experienced childhood maltreatment, to further inform intervention and prevention efforts.

### Study limitations

Several limitations of the current study should be noted. First, sampling was not random; it was based on referral and matching of elementary and middle school students, which limited the diversity of the sample in terms of racial or ethnic background and educational background. Thus, the findings may not be generalisable to adolescents who drop out of school, those with different ethnic backgrounds, those from different regions or clinical samples. A second limitation of the study was that children were the sole reporters of maltreatment and parental rejection, which may have increased the strength of observed relationships among variables because of shared-methods variance.^49^ Another probable source of bias in our study relating to retrospective self-reporting was that some abuse experiences may not have been self-identified. Thus, future research should include data from multiple reporters of abuse occurrence and parental rejection, with more comprehensive measures. Finally, all adolescents participating in this study were aged 12–16 years. Measures (i.e. randomised controlled matching) were taken to prevent age from influencing the findings. However, some behavioural problems and mental illness may not manifest until early or later adulthood. This study also has several strengths. The sample was large. It tested the traditional notion that females respond to parental rejection with internalising, rather than externalising, behaviours. Finally, the finding that paternal and maternal rejection had different mediating effects on the relationship between maltreatment and internalising behaviour in the male and female cohorts advances research on gender differences in the outcomes of childhood maltreatment.
